# Adults with intellectual disabilities and mental health disorders in primary care: a scoping review

**DOI:** 10.3399/BJGP.2021.0164

**Published:** 2021-12-14

**Authors:** Katrien PM Pouls, Monique CJ Koks-Leensen, Mathilde Mastebroek, Geraline L Leusink, Willem JJ Assendelft

**Affiliations:** Department of Primary and Community Care, Radboud University Medical Center, Nijmegen, the Netherlands.; Department of Primary and Community Care, Radboud University Medical Center, Nijmegen, the Netherlands.; Department of Primary and Community Care, Radboud University Medical Center, Nijmegen, the Netherlands.; Department of Primary and Community Care, Radboud University Medical Center, Nijmegen, the Netherlands.; Department of Primary and Community Care, Radboud University Medical Center, Nijmegen, the Netherlands.

**Keywords:** general practitioners, intellectual disability, mental disorders, mental health services, organisation and administration, primary health care

## Abstract

**Background:**

GPs are increasingly confronted with patients with both intellectual disabilities (ID) and mental health disorders (MHD). Currently, the care provided to these patients is found to be insufficient, putting them at risk of developing more severe MHD. Improving the quality of GP care will improve the whole of mental health care for this patient group. Therefore, an overview of the content and quality of care provided to them by the GP may be helpful.

**Aim:**

To provide an up-to-date literature overview of the care provided by GPs to patients with ID and MHD, identify knowledge gaps, and inform research, practice, and policy about opportunities to improve care.

**Design and setting:**

Scoping review.

**Method:**

PubMed, PsycINFO, EMBASE, and grey literature were searched for publications concerning primary care and patients with ID and MHD. Selected publications were analysed qualitatively.

**Results:**

One hundred publications met the inclusion criteria. Five overarching themes were identified: GP roles, knowledge and experience, caregiver roles, collaboration, and a standardised approach. The results show GPs’ vital, diverse, and demanding roles in caring for patients with both ID and MHD. GPs experience problems in fulfilling their roles, and gaps are identified regarding effective GP training programmes, applicable guidelines and tools, optimal collaborative mental health care, and corresponding payment models.

**Conclusion:**

The improvement required in the current quality of GP care to patients with ID and MHD can be achieved by bridging the identified gaps and initiating close collaborations between care professionals, policymakers, and organisational managers.

## INTRODUCTION

GPs are increasingly confronted with patients with both intellectual disabilities (IDs) and mental health disorders (MHDs), caused mainly by a growing need for care and support for people with mild to borderline intellectual disabilities (IQ 55–85), arising from an increasingly complex society.^1^ Patients with ID have greater healthcare needs with higher levels of morbidity and premature mortality than patients without ID,^2^^,^^3^ a situation where insufficient quality of health care is a substantial contributor.^2^ Patients with coexisting MHD form an extra vulnerable group in this context.

Although research shows that MHDs are 3–4 times more common in people with ID than in the general population,^4^^,^^5^ primary and secondary care provided to these patients is insufficient.^6^^–^^8^ This is the consequence of inadequate identification of IDs and MHDs, communication difficulties, atypical presentation of disorders, and a lack of assessment tools and treatment modalities specifically adapted to people with ID.^9^^–^^11^ But the poor accessibility to (mental) health services for patients with ID is also described as an important contributing factor.^12^^,^^13^ Consequently, patients with both ID and MHD are at risk of developing more severe or chronic MHDs, are prescribed more psychotropic medication, and use more services.^14^

In many countries, GPs are often the first care providers contacted by these patients. Their care provision constitutes both a demand-driven approach in assessment, treatment, and follow-up of symptoms and disorders, and a more proactive approach aimed at prevention by identifying risk factors and providing health education. Adequate primary care, including both of these approaches, is essential for patients with both ID and MHD to prevent diagnostic delay and ensure appropriate and timely referral and early personalised treatment.^15^

Although studies are increasingly published on expanding knowledge and skills within mental health care and ID care,^16^^,^^17^ reported practices are mostly on a small scale and lack an orientation towards primary care. This is surprising and disturbing, considering GPs’ important position in the mental healthcare system and the growing demands of this patient group.

A scoping review was conducted to enhance the level of knowledge and provide an up-to-date overview of GP care for patients with both ID and MHD, identify knowledge gaps, and inform research, practice, and policy about opportunities to improve care.

## METHOD

This review follows Arksey and O’Malley’s^18^ framework for scoping reviews, revised by Khalil *et al*,^19^ and describes methods and results in line with the PRISMA Extension for Scoping Reviews checklist.^20^

**Table:** How this fits in

The GP, as first-line healthcare professional and gatekeeper, has a vital role in the mental health care of patients with both an intellectual disability (ID) and a mental health disorder (MHD). Current GP care is considered insufficient, and the quality of care needs to be improved. However, there is a need for an overview on the care provided to these patients by GPs. This scoping review provides an up-to-date literature overview of the care provided by GPs to patients with ID and MHD, identifies knowledge gaps, and informs not only GP practice but also research and policy about ways to improve the quality of care.

### Defining the research question

The guiding research question was: what has been described about the care for adult patients with both ID and MHD provided by GPs? The key concepts are defined in Supplementary Table S1.

### Retrieving relevant publications

Relevant publications were identified using a three-step literature search.^19^ First, PubMed, PsycINFO, and EMBASE were explored (date range from January 1994 to September 2019), using search terms pertaining to ‘primary care’ and ‘intellectual disability’, informed by an information specialist (Supplementary Table S1). Selected publications were searched for keywords missed in the initial search. This yielded an additional search string for ‘ID’ (Supplementary Table S1). Second, relevant publications were retrieved from grey literature, using the recommendations from ‘Grey Matters’.^21^ Common search terms for ‘primary care’ and ‘intellectual disability’ in English and in Dutch were used (Supplementary Table S1). Third, the reference lists were searched for additional relevant publications.

### Selection of publications

Duplicates were removed, and the first author performed a first selection on title and abstract. About 20% were double screened by a second independent researcher. The full texts were then screened by the first author and a second independent researcher. Any judgement differences were discussed to reach consensus within each review pair. When consensus could not be reached, a third reviewer became involved to resolve outstanding conflicts. Publications were included if they concerned adults with an ID, an MHD, and primary care following the prepared definitions. Other criteria were:

- adult focused (≥18 years);
- originating in Western European and Anglo-Saxon countries, where GPs have a comparable role as gatekeeper for more specialised mental health care;
- available in full text; and
- available in English or Dutch.

There was no selection on publication type. Publications on forensic primary care were excluded because they concern a selective group of patients beyond the scope of this review.

### Presentation and collation of the data

A standardised data extraction form was developed to guide data charting for descriptive analysis, including publication year, country of origin, publication type, domain, and the care element(s) described. The selected publications’ content was qualitatively analysed using conventional content analysis^22^ supported by ATLAS.ti software (version 8.4). This process involved repeatedly reading the articles, identifying relevant text fragments, and inductively generating codes related to the research question. All coding was conducted by two researchers independently. Differences in coding were discussed to reach consensus. Codes were then sorted depending on how they were related. From this, major themes were developed and organised. This iterative process was followed critically by the research team, and key findings were discussed relating to the study’s purpose and implications for future research, practice, and policy.

## RESULTS

### Descriptive results

**Figure 1.**
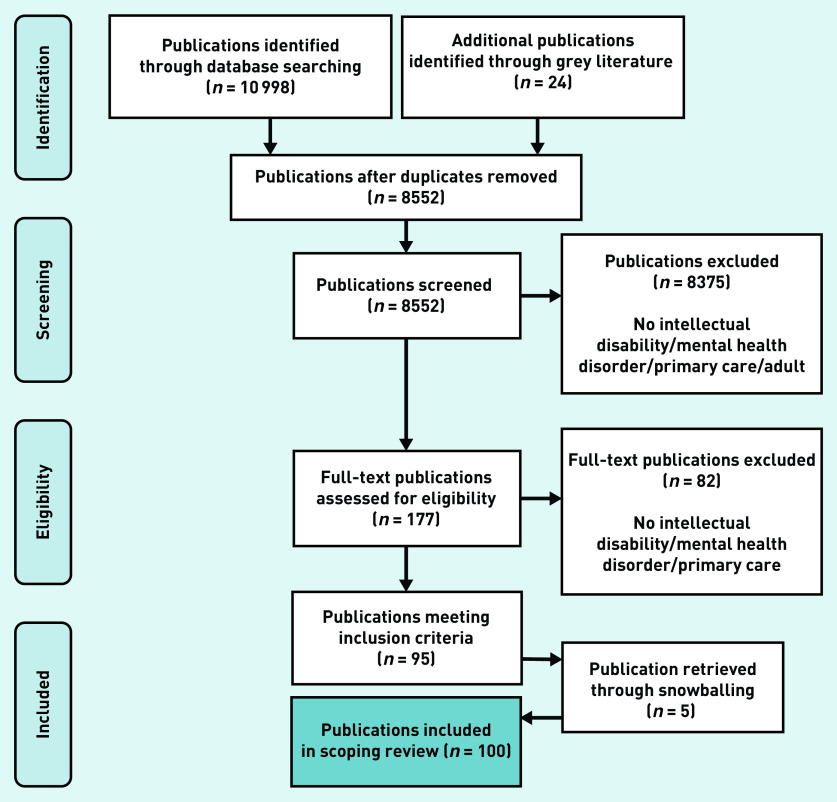
*Flow diagram of study selection process.*

**Figure 2.**
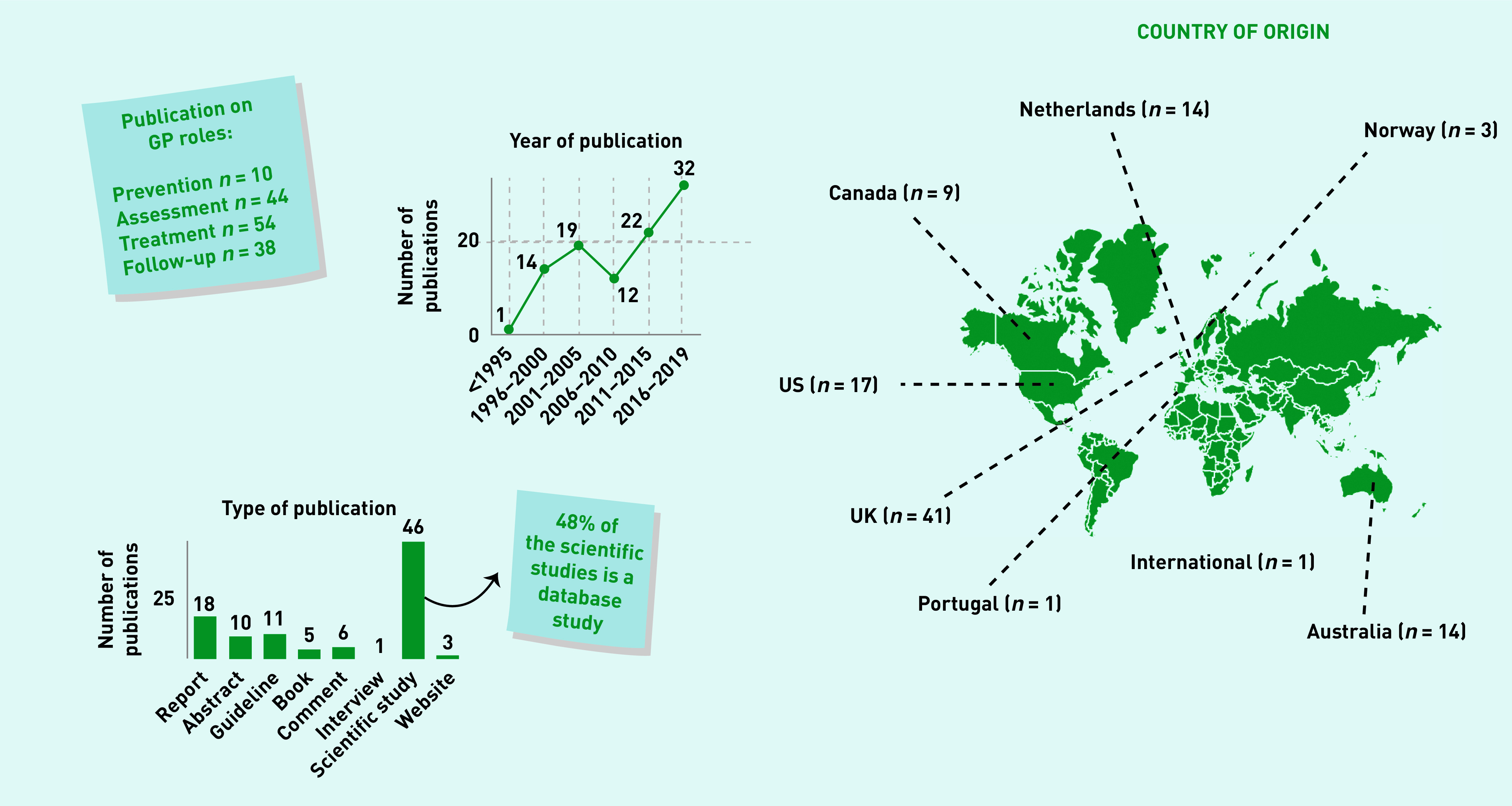
*Overview characteristics of the included publications.*

Figure 1 presents the publication selection process. One hundred publications were included for final analysis (Supplementary Table S2). The publications’ main domains were ID care (*n* = 39) and primary care (*n* = 34). The number of yield publications increases steadily over the years. In total, 46 of the publications described a scientific study, none of which were randomised controlled trials. Figure 2 presents the overview of relevant characteristics of included publications.

### Prevalence of mental health disorders in primary care

The publications on register-based cohort studies report that people with ID have a higher risk of MHDs than patients without ID.^23^^–^^26^ Primary care databases show a prevalence of MHDs in adult patients with ID ranging from 21%–34%.^4^^,^^23^^,^^25^^–^^27^ GPs’ screening of patients with ID or MHD identified 33%–71% of patients as having a possible MHD and needing further medical assessment.^28^^–^^31^ Severe mental illnesses such as schizophrenia, bipolar disorder, and psychosis are more prevalent in patients with ID.^3^^,^^4^^,^^23^^,^^32^^,^^33^ Depression and anxiety are recorded less,^33^^,^^34^ equally,^4^^,^^34^ or more often,^23^ with an earlier age of onset for depression.^35^ Smoking, alcohol, and other substance use are less prevalent,^23^^,^^33^ but alcohol misuse is more prevalent in patients with ID.^23^

### Thematic results

Thematic analysis revealed five overarching themes: GP roles, knowledge and experience, caregiver roles, collaboration, and a standardised approach. The results are narratively presented accordingly. Box 1 presents the key findings per theme.

**Box 1.** Primary care for patients with both ID and MHD

**GP roles** Prevention — General health promotion — Education on potential risk factors for MHDs — Identify provoking risk factors for MHDs and act on them Assessment — Multidimensional assessment of MHDs Treatment — Treatment of common and less complex MHDs — Referral of rare or complex MHDs — Prescribing psychotropic medication Follow-up — Monitoring treatment responses and adverse side effects — Coordinating multidisciplinary care **GPs’ knowledge and experience** Low priority in research and GPs’ educational programmes Lack of evidence-based primary care knowledge Reliance on experience-based knowledge **Caregiver roles** Recognising symptoms of MHDs and seeking help Overcoming communication difficulties Providing additional information Joint decision making Executing and monitoring the treatment plan Identifying adverse side effects of psychotropic medication **Collaboration** Forms of collaboration — Collegial advice — Handing patient over to another professional — Integrative care Preconditions for adequate collaboration — Referral options with clear procedures — Adequate information exchange — Consensus on responsibilities — Payment models as an incentive for collaboration **A standardised approach** A standardised multidimensional approach may improve the quality of care Guidelines and tools applicable to patients with ID

*ID = intellectual disability. MHD = mental health disorder.*

#### GP roles

The publications reveal the GP as the key figure in the identification, initiation, and coordination of treatment of patients with ID and MHD.^31^^,^^36^^–^^45^ Several GP roles in the care for this patient group are described, however, with varying acceptance, experience, and fulfilment among GPs.^42^^,^^46^^,^^47^

The GP roles relate, in the first place, to ‘prevention of MHDs’,^32^^,^^43^^,^^44^^,^^48^^,^^49^ in the sense of a ‘proactive approach’. This comprises general health promotion and targeted education about healthy living or substance use,^32^^,^^44^^,^^49^ but also identification of MHD-provoking risk factors and development of prevention strategies.^31^^,^^43^^,^^48^^,^^49^

Second, GPs are expected to fulfil an important role in the *diagnostic assessment of MHDs*, which is described as challenging for GPs.^50^ Indications of inaccurate diagnoses^48^^,^^51^ and underdiagnosis in primary care are frequently reported^4^^,^^31^^,^^41^^,^^42^^,^^48^^,^^49^^,^^52^^–^^56^ and may result in inappropriate care and progression of the disorder to a more severe stage that is less responsive to treatment.^23^^,^^52^^,^^57^^–^^59^ Overdiagnosis occurs as well, however, leading to unnecessary prescriptions of medication.^53^^,^^57^ Diagnostic failure is seen as related to communication problems, with patients with ID described as less able to label their feelings and communicate their needs,^44^^,^^53^^,^^57^^,^^60^^–^^62^ resulting in an atypical presentation of symptoms^29^^,^^43^^,^^44^^,^^57^^,^^63^^–^^65^ and a more complicated assessment.^42^^,^^48^^,^^52^^,^^54^^,^^61^^,^^66^ It is deemed important for the GP to exclude somatic, environmental, and other causes of symptoms before considering an MHD,^43^^,^^44^^,^^48^^,^^51^^,^^53^^,^^64^^,^^65^^,^^67^^–^^73^ which requires a holistic, multidimensional approach.^29^^,^^44^

Third, regarding GPs’ ‘treatment role’, it is indicated that GPs should be able to handle less complex MHDs in people with ID.^40^^,^^44^^,^^68^ For this patient group the same requirements and treatment guidelines apply as for patients with MHD but without ID,^44^^,^^57^^,^^65^^,^^71^^,^^73^ with the necessity to refer patients with more complex disorders to specialised care.^44^^,^^68^ GPs prescribe psychotropic medication to a higher number of patients with ID (17%– 63%) than other patients.^4^^,^^74^^,^^75^ In addition, a higher number of patients with ID are reported with psychotropic prescriptions than with recorded MHDs, indicating off-label prescription.^4^^,^^39^^,^^44^^,^^49^^,^^75^^–^^80^ Behavioural problems are often described as an indication to start medication,^4^^,^^39^^,^^59^^,^^73^^,^^74^^,^^78^^,^^79^ specifically in cases of limited access to alternative treatment strategies.^4^ Prescribed medication is reported as predominantly long-term medication,^75^ and a large proportion (62%–70%) is prescribed without a psychiatrist’s involvement.^50^^,^^51^^,^^81^ Medication prescription can be considered part of a multidisciplinary and holistic care plan;^65^ however, in primary practice, GPs are less likely than psychiatrists to provide psychosocial interventions.^72^

Finally, ‘follow up’ by GPs is considered an essential element in the treatment of patients with ID and MHD.^48^^,^^50^^,^^65^^,^^69^^,^^73^^,^^82^^–^^84^ It enables the monitoring of treatment responses and possible adverse side effects, leading to early adjustment of the treatment plan.^50^^,^^73^ However, a lack of effective monitoring of psychotropic medication by GPs is often described.^28^^,^^42^^,^^51^^,^^55^^,^^74^^,^^81^^,^^85^^,^^86^ It is stipulated that this relates to GPs’ lack of specific experience and knowledge,^51^^,^^81^^,^^87^ uncertainty about who of the involved professionals is responsible for follow-up,^47^^,^^83^^,^^86^^,^^88^^,^^89^ patient problems in communicating and presenting (side) effects of medication,^44^^,^^49^^,^^65^ and dependence on observations by, and cooperation with, caregivers.^39^^,^^44^^,^^49^^,^^50^^,^^62^^,^^69^^,^^72^

#### Knowledge and experience

There is general consensus that GPs generally have limited knowledge about, and experience in, managing patients with ID and MHD,^24^^,^^37^^,^^40^^,^^42^^,^^43^^,^^46^^,^^50^^–^^52^^,^^58^^,^^66^^,^^69^^,^^81^^,^^87^^,^^90^^–^^96^ caused by a lack of priority in medical training programmes^37^^,^^40^^,^^43^^,^^50^^,^^52^^,^^58^^,^^66^^,^^93^ and a lack of research concerning this patient group.^93^ This results in limited evidence-based knowledge^43^^,^^47^^,^^50^^,^^65^^,^^67^^,^^93^^,^^97^ and reliance on experience-based knowledge instead.^50^ It is indicated that limited knowledge and experience create feelings of insecurity in GPs when addressing patients with ID and MHD,^50^ lack of caregivers’ confidence in the GP,^52^ and insufficient care.^37^^,^^41^^,^^44^^,^^52^^,^^58^^,^^66^^,^^81^^,^^91^^,^^92^^,^^97^ Although GPs are interested in more training and education regarding patients with ID and MHD,^24^^,^^37^^,^^41^^,^^42^^,^^44^^,^^52^^,^^58^^,^^66^^,^^87^^,^^90^^,^^91^ in practice it is seen as a challenge to engage them, caused by the small size of the population and the variety of competing medical issues.^58^^,^^70^ Publications underline the importance of investment in more research and initiatives for effective training, skill development,^37^^,^^42^^,^^50^^,^^58^^,^^92^^,^^96^ and evidence-based guidelines for GPs.^42^^,^^50^^,^^98^

#### Caregiver roles

In the publications, it is noted that patients with ID and MHDs are often reliant on formal or informal caregivers for receiving care,^23^^,^^43^^,^^49^^,^^57^^,^^62^^,^^69^^,^^72^^,^^99^ as a first point of reference, to recognise symptoms of MHDs and seek help.^23^^,^^43^^,^^62^^,^^73^^,^^99^ For this task, it is deemed important that caregivers have some knowledge of associated symptoms; however, this knowledge is often lacking.^23^^,^^42^^–^^44^^,^^100^ Second, patients are frequently dependent on caregivers for joint decision making^44^ and giving informed consent regarding treatment options such as psychotropic medication.^4^^,^^49^^,^^65^^,^^68^^,^^72^ Third, caregivers have important roles in the execution of the treatment plan regarding medication adherence and identifying and monitoring possible side effects.^49^^,^^50^^,^^73^^,^^82^ A symptoms checklist is mentioned as a helpful tool for caregivers to provide the GP with the information needed.^40^^,^^73^ Furthermore, it is noted that the referral process can be complex, and support by caregivers can be essential to prevent delay in care.^96^^,^^100^

In addition, the GP is reliant on caregivers understanding symptom presentation,^29^^,^^43^^,^^44^^,^^53^^,^^57^^,^^60^^–^^65^ overcoming communication difficulties,^43^^,^^57^^,^^69^^,^^72^ and providing additional information.^57^^,^^63^^,^^73^ It is indicated that the more severe the ID, the more reliant the GP is on caregivers.^63^ Therefore, GPs should determine the key people in a patient’s life^73^ and proactively involve them.^49^^,^^72^ However, GPs should also realise that some caregivers may give information from their personal perspective, use different definitions of medical terms than the GP,^44^ and themselves have limited knowledge about the patient^53^^,^^62^^,^^72^^,^^81^^,^^94^ or limited communication skills.^42^^,^^44^

#### Collaboration

The publications emphasise the importance of GPs collaborating with other professionals in providing care for patients with ID and MHD. The collaborative partners mentioned are diverse and comprise both medical specialists (for example, psychiatrists and pharmacists) and services such as community or addiction services. Described areas for collaboration are the assessment of symptoms,^43^^,^^44^^,^^49^^,^^50^^,^^56^^,^^63^^,^^64^^,^^72^^,^^75^ level of communicational skills and cognitive functioning,^44^^,^^86^ and obtaining advice on referral,^31^^,^^72^^,^^78^ treatment,^30^^,^^44^^,^^48^^–^^50^^,^^72^^,^^75^^,^^86^ or prevention.^49^ GPs’ options include referral for collegial advice,^31^^,^^48^^,^^72^^,^^94^ handing the patient over to other professionals,^44^ or joining a multidisciplinary team giving integrative care to the patient.^93^^,^^98^^,^^101^ The latter is described as particularly desirable when the patient has a more severe ID or is in a highly complex situation.^39^^,^^44^^,^^99^

Effective collaboration is seen as beneficial for the outcome of mental health care in primary care^28^^,^^39^^,^^42^^,^^44^^,^^93^^,^^94^^,^^96^^,^^98^^,^^102^ as it is believed to increase the identification of MHDs, improve access to mental health care,^98^^,^^101^ and reduce hospitalisation^103^ and costs.^98^^,^^101^ However, inappropriate referrals are reported,^90^^,^^94^ resulting from unclear referral options and procedures.^44^^,^^45^^,^^96^^,^^100^^,^^104^ Adequate information exchange between GPs and other professionals, in the form of standardised, timely letters, is underlined as important for sharing essential information^38^^,^^62^^,^^94^^,^^105^^–^^107^ and continuity of care.^105^^,^^106^ Yet, audit studies on referral letters and letters from psychiatrists to GPs show that important information is often missing.^62^^,^^94^^,^^100^^,^^105^^,^^107^ Finally, it is stated that, in multidisciplinary collaboration, the alignment of responsibilities in treatment and follow-up should be clear.^44^^,^^68^^,^^108^ Particularly in cross-domain collaboration, it may be unclear who is involved, how responsibilities are shared, and how care is financed.^47^^,^^83^^,^^86^^,^^88^^,^^89^^,^^96^^,^^104^ It is mentioned that adequate division of responsibilities may depend on the main causal factor(s) of the MHD and may necessitate using care plans and convening case conferences.^68^ Responsibilities should be clearly stated in writing and reviewed regularly.^88^ Consequently, suggested preconditions for effective collaboration are accessible referral options, clarity about referral procedures, adequate information exchange between the professionals, and consensus on responsibilities.^3^^,^^24^^,^^39^^,^^41^^,^^88^^,^^94^^,^^96^^,^^107^

It is noted as a barrier that, from a historical point of view, primary and secondary care services are separate units culturally,^98^ organisationally,^42^ and financially.^96^^,^^98^^,^^101^ To improve the quality of collaboration, the roles of both GPs and other involved professionals should be defined more clearly,^60^^,^^72^ existing models should be evaluated,^42^^,^^109^ clinical pathways and/or models should be improved,^24^^,^^42^^,^^109^ specialist capacity should be enhanced,^41^ and payment models should be re-examined to stimulate collaborative care.^98^^,^^101^ Policymakers’ involvement in this matter is seen as important.^98^^,^^101^

#### A standardised approach

In several publications, a standardised approach is seen as a way to improve the quality of care for patients with both ID and MHD.^43^^–^^45^^,^^49^^,^^50^^,^^65^^,^^68^^,^^83^^,^^110^ First, standardised screening for MHDs gives GPs the opportunity to consider potential mental health issues at an early stage.^44^^,^^49^ Second, a structured multidimensional approach in the assessment leads to more appropriate and accurate diagnosis, treatment, and referral.^44^^,^^45^^,^^49^^,^^65^^,^^68^ Finally, systematic and standardised medication prescriptions and reviews identify potential medication-related problems at an early stage.^43^^,^^50^^,^^65^^,^^83^^,^^110^

Although guidelines and instruments are available to support GPs in applying a standardised approach in the general population, they are often not adapted to patients with ID.^40^^,^^43^^,^^44^^,^^72^^,^^73^^,^^80^ Some publications covered specific guidelines for prescribing and/or monitoring medication for MHDs in patients with ID,^39^^,^^47^^,^^49^^,^^54^^,^^59^^,^^65^^,^^67^^,^^73^^,^^82^ and applicable tools for detecting unmet health needs in patients with ID.^31^^,^^40^^,^^49^^,^^60^^,^^69^ It is suggested that GPs are insufficiently familiar with these ID-specific guidelines and tools.^51^^,^^87^

## DISCUSSION

### Summary

To the authors’ knowledge, this is the first scoping review with a focus on patients with both ID and MHD in primary health care. GPs are a key figure in the care for this specific patient group. They have a broad role that can be demanding in the sense that GPs need specific knowledge, experience, and skills for a relatively small patient group. The publications indicate that current GP care is often of an insufficient standard, as reflected in underdiagnosis of MHDs, overmedication, and lack of effective patient follow-up. Gaps are identified regarding effective training programmes for GPs, applicable guidelines and tools, optimal collaborative mental health care, and corresponding payment models. Opportunities for improvement are seen in prioritising and investing in bridging these gaps. This requires the involvement not only of care professionals and scientists, but also of policymakers.

### Strengths and limitations

The first strength of this review is that a robust and widely accepted scoping review method^19^ was used to provide a solid overview of the existing knowledge on GP care for patients with both ID and MHD. Second, only publications from countries where GPs have a gatekeeper role were included. This results in recommendations that can improve not only the quality of GP care, but also the overall mental health care for patients with ID. Finally, to prevent dispersion of the results of an already broad research question, this study focused on adults, thereby giving attention to a vulnerable group that is potentially more overlooked than children.

This review also has limitations. First, a lack of consistency was found in the definition of ID, as many publications did not supply a clear definition of it. This limitation reflects the heterogeneity of the patient group, and results should be interpreted accordingly. Second, the publications retrieved in the grey literature search are presumably not perfectly complete, despite the use of the ‘Grey Matters’ tool^21^ and checking references lists. Some publications were not accessible for the research team, and publications could have been missed because of the great diversity of possible sources.

### Comparison with existing literature

The results of this review indicate that patients with ID constitute a small group within the GP population, accounting for the low priority of this group in education and research.^58^^,^^70^ However, this claim regarding the proportion of patients is debatable. In the Netherlands, it is estimated that 6.4% of the population have a mild ID.^1^ Research in primary care data shows that no more than 0.56% of GPs’ clients are registered as having an ID.^23^^,^^34^ This could be an indication of GPs’ insufficient recognition and underestimation of the size of the ID population. This underestimation is also present in mental health care and is a reason for concern.^7^ Identification of an ID is essential both for good care provision and for treatment success in MHDs.^14^ Helpful ID screening tools have been developed for GPs when they are considering an ID, but further implementation in practice is needed.^111^^,^^112^

The results from this study revealed two strategies to improve care for patients with both ID and MHD: adequate medical training and applicable evidence-based guidelines and tools. This is in line with previous reports and publications concerning general health issues in patients with ID.^12^^,^^113^ It is also suggested that GPs should use the same treatment guidelines for mild or less complex MHDs in patients either with or without ID.^44^^,^^57^^,^^65^^,^^71^^,^^73^ However, research to substantiate this is scarce, and research in addiction care shows that alterations in the treatment programmes for substance use disorders are needed for patients with ID.^10^ Furthermore, previous research projects have led to practical primary care tools, such as the Psychiatric Assessment Schedule for Adults with Developmental Disabilities (PAS-ADD).^31^^,^^60^ However, these tools are not fully implemented in primary care.

This review identified various kinds of primary care collaborations in which GPs participate, and shows that effective collaboration can improve care; the latter is widely supported and confirmed by the World Health Organization.^114^ This review had an international focus, and the possibilities of multidisciplinary approaches in primary care differ in the various healthcare systems. Within the ambitions and possibilities of the NHS, the UK sees integrated care systems as an important tool for improving health care and for reducing inequalities between different groups of people,^115^ and has long-term experience with community learning disability teams. These multidisciplinary teams provide health care and advice to people with ID, GPs, carers, families, and to the wider health and social care community.^116^ Another example of promising collaboration in daily practice is ‘The DD Health Home’ in the US. This care model provides integrative routine care to patients with ID and MHD, including primary care and structural follow-up.^98^ Despite these best practices, there is at present limited scientific evidence on the effectiveness of these collaborative (mental) healthcare services for persons with ID.^117^ Preconditions for collaborative care, as listed in Box 1, are also recognised in older people and chronic disease care.^118^^–^^120^ All these disciplines mention adequate reimbursement as a critical barrier to successful collaboration, and reimbursement needs to be prioritised. Additionally, collaboration is more effective when there is a team vision, shared goals, formal quality processes, and shared ICT information systems.^118^^,^^120^ Research in older person care has revealed that GPs are indispensable in multidisciplinary teams regarding networking activities, integration of care, and showing leadership; the researchers stress that GPs should be made more aware of this, for instance, in GP training programmes.^121^

Finally, the results of this review stress the importance of the GP collaborating with caregivers. Remarkably, none of the included publications focused on patients’ needs in their contact with the GP, although research shows that people with ID prefer to be less reliant on caregivers in GP consultation and argue for an improvement of the accessibility of health services.^13^^,^^122^^,^^123^ The UK National Institute for Health and Care Excellence guideline *Mental Health Problems In People With Learning Disabilities: Prevention, Assessment And Management* gives special attention to the involvement of people with ID and their caregivers in organising their care. In addition, this guideline covers mental healthcare in a holistic way in different UK settings, and may serve as an example for other countries.^124^

### Implications for research and practice

Improvement of care for patients with both ID and MHD needs to be prioritised, justified by the limited quality of care and the substantial size of this patient group. This improvement cannot be achieved by GPs and their collaborative partners alone. It requires adaptations on both the organisational and the political level. However, it remains important to actively engage GPs to ensure that suggested strategies are applicable and feasible in daily practice.

Diverse improvement strategies are advisable. First, it is important to invest in effective, frequently recurring post-curriculum training programmes for GPs, focusing on more awareness of this patient group, specific knowledge gaps regarding IDs and MHDs, existing tools and guidelines, and GP roles in multidisciplinary teams. Offering training programmes in an interprofessional setting can support the latter and will additionally promote collaboration between care professionals. Second, ongoing policy changes, such as the move to integrated care systems in the UK, and best practices such as the community learning disability teams in the UK, provide opportunities for further development of optimal collaborative healthcare models for patients with both ID and MHD. Policymakers should re-examine payment models to create incentives for collaborative care, facilitate shared ICT information facilities, and involve potential users in the development of these healthcare models. Third, although the increase in publication over the recent years is a positive sign, more research is still needed on the effectiveness of existing general mental health guidelines and tools to determine whether they are truly applicable to patients with ID or whether specific alterations are needed. Priority should be given to guidelines and tools on MHD assessment, the prescription of psychotropic medication, and patient follow-up. Finally, further research should focus on the needs of caregivers in supporting patients, as well as on the needs of patients with both ID and MHD and how they can enhance their autonomy in GP contacts.

This scoping review illustrates GPs’ vital roles in the care of patients with both ID and MHD. Current GP care has generally proved insufficient, and improvement strategies are needed in close collaboration with policymakers and organisational managers. Multidisciplinary approaches in primary care — like those in the UK and US — seem promising, but still lack sufficient scientific evaluation. Investment in education, evidence-based guidelines and tools, and collaborative healthcare models is essential. This, supplemented with enhanced ID identification and attention to the needs of patients and their caregivers, may significantly improve the quality of care for this vulnerable patient group.

## Data

Qualitative data are available from the corresponding author upon reasonable request.

## References

[1] Woittiez I, Eggink E, Ras M (2019). [The number of people with a mild intellectual disability: an estimate]. [In Dutch]. Den Haag: Sociaal en Cultureel Planbureau.

[2] Heslop P, Blair PS, Fleming P (2014). The Confidential Inquiry into premature deaths of people with intellectual disabilities in the UK: a population-based study. Lancet.

[3] Carey IM, Hosking FJ, DeWilde S (2016). Learning disability registers in primary care. Br J Gen Pract.

[4] Sheehan R, Hassiotis A, Walters K (2015). Mental illness, challenging behaviour, and psychotropic drug prescribing in people with intellectual disability: UK population based cohort study.. BMJ.

[5] Cooper SA, Smiley E, Morrison J (2007). Mental ill-health in adults with intellectual disabilities: prevalence and associated factors. Br J Psychiatry.

[6] Bertelli MO, Rossi M, Scuticchio D, Bianco A (2015). Diagnosing psychiatric disorders in people with disabilities: issues and achievements. Adv Ment Health Intellect Disabil.

[7] Seelen-de Lang BL, Smits HJH, Penterman BJM (2019). Screening for intellectual disabilities and borderline intelligence in Dutch outpatients with severe mental illness. J Appl Res Intellect Disabil.

[8] Van Duijvenbode N, VanDerNagel JEL, Didden R (2015). Substance use disorders in individuals with mild to borderline intellectual disability: current status and future directions.. Res Dev Disabil.

[9] Manohar H, Subramanian K, Kandasamy P (2016). Diagnostic masking and overshadowing in intellectual disability — how structured evaluation helps. J Child Adolesc Psychiatr Nurs.

[10] Kiewik M, VanDerNagel JEL, Engels R, De Jong CAJ (2017). Intellectually disabled and addicted: a call for evidence based tailor-made interventions. Addiction.

[11] Lindsay P, Burgess D (2006). Care of patients with intellectual or learning disability in primary care: no more funding so will there be any change?. Br J Gen Pract.

[12] Krahn GL, Hammond L, Turner A (2006). A cascade of disparities: health and health care access for people with intellectual disabilities. Ment Retard Dev Disabil Res Rev.

[13] Burke CK (2014). Feeling down. Improving the mental health of people with learning disabilities.

[14] Hassiotis A, Strydom A, Hall I (2008). Psychiatric morbidity and social functioning among adults with borderline intelligence living in private households. J Intellect Disabil Res.

[15] Hansen J, Groenewegen PP, Boerma WG, Kringos DS (2015). Living in a country with a strong primary care system is beneficial to people with chronic conditions. Health Aff (Millwood).

[16] Van Duijvenbode N, VanDerNagel JEL (2019). A systematic review of substance use (disorder) in individuals with mild to borderline intellectual disability. Eur Addict Res.

[17] Whittle EL, Fisher KR, Reppermund S, Trollor J (2019). Access to mental health services: the experiences of people with intellectual disabilities. J Appl Res Intellect Disabil.

[18] Arksey H, O’Malley L (2005). Scoping studies: towards a methodological framework. Int J Soc Res Methodol.

[19] Khalil H, Peters M, Godfrey CM (2016). An evidence-based approach to scoping reviews. Worldviews Evid Based Nurs.

[20] Tricco AC, Lillie E, Zarin W (2018). PRISMA extension for scoping reviews (PRISMA-ScR): checklist and explanation. Ann Intern Med.

[21] Canadian Agency for Drugs and Technologies in Health (2019). Grey Matters: a practical tool for searching health-related grey literature.

[22] Hsieh HF, Shannon SE (2005). Three approaches to qualitative content analysis. Qual Health Res.

[23] Cooper SA, McLean G, Guthrie B (2015). Multiple physical and mental health comorbidity in adults with intellectual disabilities: population-based cross-sectional analysis. BMC Fam Pract.

[24] Centre for Addiction and Mental Health (2013). Enhancing mental health care across the lifespan for Ontarians with developmental disabilities.

[25] Van Schrojenstein Lantman-de Valk H, Jabaaij L (2006). [People with intellectual disabilities require more care from their GP]. [In Dutch]. Huisarts Wet.

[26] Van Schrojenstein Lantman-de Valk H, te Wierik M, van den Akker M (2004). Morbidity and health-care use in people with intellectual disabilities in general practice: first results of a survey in the Netherlands. J Policy Pract Intellect Disabil.

[27] Van Schrojenstein Lantman-de Valk H, Metsemakers J, Haveman M, Crebolder H (2000). Health problems in people with intellectual disability in general practice: a comparative study. Fam Pract.

[28] Cassidy G, Martin DM, Martin GHB, Roy A (2002). Health checks for people with learning disabilities: community learning disability teams working with general practitioners and primary health care teams. J Learn Disabil.

[29] Felce D, Kerr M, Hastings RP (2009). A general practice-based study of the relationship between indicators of mental illness and challenging behaviour among adults with intellectual disabilities. J Intellect Disabil Res.

[30] Martin BA (1997). Primary care of adults with mental retardation living in the community. Am Fam Physician..

[31] Roy A, Martin DM, Wells MB (1997). Health gain through screening — mental health: developing primary health care services for people with an intellectual disability. J Intellect Dev Disabil.

[32] Cooper SA, Hughes-McCormack L, Greenlaw N (2017). Management and prevalence of long-term conditions in primary health care for adults with intellectual disabilities compared with the general population: a population-based cohort study.. J Appl Res Intellect Disabil.

[33] McDermott S, Platt T, Krishnaswami S (1997). Are individuals with mental retardation at high risk for chronic disease?. Fam Med.

[34] Carey IM, Shah SM, Hosking FJ (2016). Health characteristics and consultation patterns of people with intellectual disability: a cross-sectional database study in English general practice. Br J Gen Pract.

[35] McDermott S, Moran R, Platt T (2005). Depression in adults with disabilities, in primary care. Disabil Rehabil.

[36] Birch RC, Cohen J, Trollor JN (2017). Fragile X-associated disorders: don’t miss them. Aust Fam Physician.

[37] Day K, Bouras N (1999). Professional training in the psychiatry of mental retardation in the UK. Psychiatric and behavioural disorders in developmental disabilities and mental retardation.

[38] Markar TN (2002). Communications between psychiatrists and general practitioners in learning disability: a clinical audit. Int J Dev Disabil.

[39] Regi TA, Shankar R, Cooper SA (2017). Challenges and pitfalls of antipsychotic prescribing in people with learning disability. Br J Gen Pract.

[40] Torr J, Iacono T, Graham MJ, Galea J (2008). Checklists for general practitioner diagnosis of depression in adults with intellectual disability. J Intellect Disabil Res.

[41] Trollor J, Salomon C (2016). Unnecessary psychotropic drug prescription in primary care for people with intellectual disability. Evid Based Ment Health.

[42] Brooks D (2001). Primary care and mental health needs. Tizard Learning Disability Review.

[43] Curran J, Hollins S (1996). The prevention of mental illness in people with learning disability The prevention of mental illness in primary care.

[44] GGZstandaarden (2018). [Generic module: Mental disorders and borderline or mild intellectual disability]. [In Dutch]. https://www.ggzstandaarden.nl/generieke-modules/psychische-stoornissen-en-zwakbegaafdheid-zb-of-lichte-verstandelijke-beperking-lvb/introductie.

[45] Slater H, Kerr M, Blake P (2017). Assessment in primary care.

[46] Lennox G, Diggens J, Ugoni A (2000). Health care for people with an intellectual disability: general practitioners’ attitudes, and provision of care. J Intellect Dev Disabil.

[47] Shankar R, Wilcock M (2018). Improving knowledge of psychotropic prescribing in people with intellectual disability in primary care. PLoS One.

[48] Baldor R (2019). Primary care of the adult with intellectual and developmental disabilities.

[49] Sullivan WF, Diepstra H, Heng J (2018). Primary care of adults with intellectual and developmental disabilities. 2018 Canadian consensus guidelines. Can Fam Physician.

[50] Fredheim T, Haavet OR, Danbolt LJ (2013). Intellectual disability and mental health problems: a qualitative study of general practitioners’ views. BMJ Open.

[51] Holden B, Gitlesen JP (2004). Psychotropic medication in adults with mental retardation: prevalence, and prescription practices. Res Dev Disabil.

[52] McGillivray JA, Kershaw MM (2013). The impact of staff initiated referral and intervention protocols on symptoms of depression in people with mild intellectual disability. Res Dev Disabil.

[53] Silka VR, Hurley AD (2003). Differentiating psychiatric and medical problems in patients with developmental disabilities. Mental Health Aspects of Developmental Disabilities.

[54] Developmental Disabilities Primary Care Initiative (2019). Health care for adults with intellectual and developmental disabilities. Toolkit for primary care providers.

[55] McCoubrie M, Baines E (2011). Primary care and intellectual disability. Advice for medical students and GPs.

[56] Lennox N, Eastgate G (2004). Adults with intellectual disability and the GP. Aust Fam Physician.

[57] Anderson E, Dive L, Kang S (2008). Family physician guide. For depression, anxiety disorders, early psychosis and substance use disorders..

[58] Costello H, Holt G, Cain N, Bouras N, Holt G (2007). Professional training for those working with people with intellectual disabilities and mental health problems. Psychiatric and behavioural disorders in intellectual and developmental disabilities.

[59] Salomon C, Britt H, Pollack A, Trollor J (2018). Primary care for people with an intellectual disability — what is prescribed? An analysis of medication recommendations from the BEACH dataset. BJGP Open.

[60] Martin DM, Roy A, Wells MB (1997). Health gain through health checks: improving access to primary health care for people with intellectual disability. J Intellect Disabil Res.

[61] Straetmans J, van Schrojenstein Lantman-de Valk H, Schellevis FG, Dinant G-J (2007). Health problems of people with intellectual disabilities: the impact for general practice. Br J Gen Pract.

[62] Taylor SC, Markar TN (2002). Audit of the quality of general practitioner referral letters to a learning disability service. Int J Dev Disabil.

[63] Messinger-Rapport BJ, Rapport DJ (1997). Primary care for the developmentally disabled adult. J Gen Intern Med.

[64] Centre for Developmental Disability Studies (2006). Health care in people with intellectual disability; guidelines for general practitioners.

[65] Royal College of Psychiatrists (2016). Psychotropic drug prescribing for people with intellectual disability, mental health problems and/or behaviours that challenge: practice guidelines..

[66] Phillips A, Morrison J, Davis RW (2004). General practitioners’ educational needs in intellectual disability health. J Intellect Disabil Res.

[67] Bakker-van Gijssel EJ, Leusink GL (2015). [Psychotropic drug prescription to people with intellectual disability in GP practices]. (In Dutch). Ned Tijdschr Geneeskd.

[68] Davis R, Mohr C (2004). The assessment and treatment of behavioural problems. Aust Fam Physician.

[69] Green L, McNeil K, Korossy M (2018). HELP for behaviours that challenge in adults with intellectual and developmental disabilities. Can Fam Physician.

[70] Munden AC, Perry DW (2002). Symptoms of depression in people with learning disabilities. J Learn Disabil.

[71] Prater CD, Zylstra RG (2006). Medical care of adults with mental retardation. Am Fam Physician.

[72] Woods R (2011). Behavioural concerns — assessment and management of people with intellectual disability. Aust Fam Physician.

[73] Tracy J, Davis R, Macgibbon P, Graham M (2015). Medication review guide for GPs. A guide for GPs on the use of psychoactive medications for adults with intellectual disability who present with behaviours of concern.

[74] Doan TN, Lennox NG, Taylor-Gomez M, Ware RS (2013). Medication use among Australian adults with intellectual disability in primary healthcare settings: a cross-sectional study. J Intellect Dev Disabil.

[75] Glover G, Williams R (2015). Prescribing Of Psychotropic Drugs To People With Learning Disabilities And/Or Autism By General Practitioners In England.

[76] Adams D (2016). Pharmacists’ role in optimising the use of medicines in those with learning disabilities. Pharm J.

[77] Glover G, Williams R, Branford D (2016). Psychotropic prescribing by general practitioners for people with intellectual and developmental disabilities in England. J Intellect Disabil Res.

[78] Molyneaux P, Emerson E, Caine A (1999). Prescription of psychotropic medication to people with intellectual disabilities in primary health-care settings. J Appl Res Intellect Disabil.

[79] Sheehan R, Horsfall L, Walters K (2015). Psychotropic prescribing for people with intellectual disability in UK primary care. J Intellect Disabil Res.

[80] Van Dijk L, Francke A (2004). [Fact sheet 6: Prescribing psycholeptics for people living at home with intellectual disabilities]. [In Dutch]. Nivel.

[81] Mahmood A (2000). GPs should not hand out medication indiscriminately to people with learning disabilities. Nurs Times.

[82] NHS England (2017). Stopping over medication of people with a learning disability, autism or both.

[83] Baburaj R, El Tahir M (2011). Monitoring for metabolic syndrome in people with intellectual disability on antipsychotic medication. Adv Ment Health Intellect Disabil.

[84] Sheehan R, Horsfall L, Strydom A (2017). Movement side effects of antipsychotic drugs in adults with and without intellectual disability: UK population-based cohort study. BMJ Open.

[85] Hilty DM, Ingraham RL, Yang SP, Anders TF (2004). Multispecialty telephone and e-mail consultation for patients with developmental disabilities in rural California. Telemed J E Health.

[86] Boot F, Mulder-Wildemors L, Voorbrood V, Evenhuis H (2018). [Medication assessment in people with intellectual disabilities.]. [In Dutch]. Huisarts Wet.

[87] Gomes T, Khuu W, Tadrous M (2019). Antipsychotic initiation among adults with intellectual and developmental disabilities in Ontario: a population-based cohort study. BMJ Open.

[88] Buckley C, Sharrard H (2003). Lithium monitoring for patients with learning disability: the role of the general practitioner. Qual Prim Care.

[89] Shankar R, Wilcock M (2019). Improving psychotropic medication prescribing in people with intellectual disability in UK primary care: an educational intervention. J Intellect Disabil Res.

[90] Ajaz A, Eyeoyibo M (2011). Referral patterns to mental health of intellectual disability team. Adv Ment Health Intellect Disabil.

[91] Bouras N, Holt G, Došen A, Day K (2001). Psychiatric treatment in community care. Treating mental illness and behavior disorders in children and adults with mental retardation.

[92] Cohen J, Loesch DZ (1999). Fragile X syndrome: do professionals know about it?. Med J Aust.

[93] Dykens EM, Hodapp RM, Fidler DJ (2016). Psychiatric disorders in people with intellectual disabilities: steps toward eliminating research and clinical care disparities. Fifty years of research in intellectual and developmental disabilities.

[94] Michael DM, Bhaumik S, Nadkarni S (2004). Misplaced or displaced? An audit of referred patients to an adult learning disability psychiatric service. Int J Dev Disabil.

[95] Bekkema N, De Veer A, Francke A (2014). [Concerns about patients with intellectual disabilities]. [In Dutch]. Huisarts Wet.

[96] Lubbes E, Pansier-Mast L, Schutte S (2019). [Specific client groups in the mental healthcare waiting time approach. Report of the research into factors and solutions]. [In Dutch]. Landelijke Stuurgroep Wachttijden GGZ.

[97] Boggon R, Glover G, Ridge K, Avery R (2014). Use of psychotropic medication for people with learning disability or autism in England: a descriptive study using the Clinical Practice Research Datalink. Pharmacoepidemiol Drug Saf.

[98] Ervin DA, Williams A, Merrick J (2014). Primary care: mental and behavioral health and persons with intellectual and developmental disabilities. Front Public Health.

[99] Maitland CH, Tsakanikos E, Holt G, Bouras N (2006). Mental health service provision for adults with intellectual disability: sources of referrals, clinical characteristics and pathways to care. Prim Care Ment Health.

[100] Van der Nagel J, van Dijk M, Kemna L (2017). [Recognized and treated correctly; guidelines for the implementation and execution of an MID-friendly intake in addiction care]. [In Dutch]. Tactus, Avelijn, Onderzoeksinstituut IVO.

[101] Ervin DA, Williams A, Merrick J (2015). Adults, mental illness and disability. Int J Disabil Hum Dev.

[102] Ferreira M, Figueira I, Silva C (2015). Dual diagnosis. Characteristics, diagnosis and trends. J Intellect Disabil Res.

[103] Balogh R, Brownell M, Ouellette-Kuntz H, Colantonio A (2010). Hospitalisation rates for ambulatory care sensitive conditions for persons with and without an intellectual disability — a population perspective. J Intellect Disabil Res.

[104] Bernard S, Bates R (1994). The role of the psychiatrist in learning disability: how it is perceived by the general practitioner. Psychiatr Bull.

[105] Markar TN, Mahadeshwar S (1998). Audit on communication between general practitioners and psychiatrists following an initial outpatient assessment of patients with learning disabilities. Br J Dev Dis.

[106] Mehnaz S, Karki C, Mukhopadhyay A (2010). Quality of psychiatrists’ letters to general practitioners on learning disabilities: GPs make their choice. Int J Dev Disabil.

[107] Thalayasingam S, Alexander RT, Singh I (1999). Audit on letters from psychiatrists to general practitioners following assessment of patients with learning disabilities in follow-up clinics. Int J Dev Disabil.

[108] NVAVG, LHV (2017). [Guidelines for cooperation between the GP and the AVG]. [In Dutch]. https://nvavg.nl/wp-content/uploads/2016/02/handreiking_huisarts_avg_2017.pdf.

[109] Chandler S, Boss K, McEntee K (2012). Collaborative mental health care for adults with intellectual disabilities: moving from policy to practice. J Intellect Disabil Res.

[110] Granas AG, Mohammed A, Aanensen JW (2017). Medication review in patients with mental disabilities. Int J Clin Pharm.

[111] Nijman H, Kaal H, van Scheppingen L, Moonen X (2018). Development and Testing of a Screener for Intelligence and Learning Disabilities (SCIL). J Appl Res Intellect Disabil.

[112] Hayes SC (2000). Hayes Ability Screening Index (HASI), manual.

[113] Heslop P, Blair P, Fleming P (2013). Confidential Inquiry into premature deaths of people with learning disabilities (CIPOLD) Final report.

[114] World Health Organization (2010). Framework for action on interprofessional education & collaborative practice.

[115] NHS (2019). The NHS Long Term Plan.

[116] Health NKC (2020). Community learning disability team.

[117] Balogh R, McMorris CA, Lunsky Y (2016). Organising healthcare services for persons with an intellectual disability.. Cochrane Database Syst Rev.

[118] Hoedemakers M, Marie Leijten FR, Looman W (2019). Integrated care for frail elderly: a qualitative study of a promising approach in the Netherlands. Int J Integr Care.

[119] Holterman S, Lahr M, Wynia K (2020). Integrated care for older adults: a struggle for sustained implementation in Northern Netherlands. Int J Integr Care.

[120] Mulvale G, Embrett M, Razavi SD (2016). ‘Gearing Up’ to improve interprofessional collaboration in primary care: a systematic review and conceptual framework. BMC Fam Pract.

[121] Grol SM, Molleman GRM, Kuijpers A (2018). The role of the general practitioner in multidisciplinary teams: a qualitative study in elderly care. BMC Fam Pract.

[122] Mastebroek M, Naaldenberg J, van den Driessen Mareeuw FA (2016). Experiences of patients with intellectual disabilities and carers in GP health information exchanges: a qualitative study. Fam Pract.

[123] Murphy J (2006). Perceptions of communication between people with communication disability and general practice staff. Health Expect.

[124] National Institute for Health and Care Excellence (2016). Mental health problems in people with learning disabilities: prevention, assessment and management NG54.

